# Correction of Femoral Torsional Deformities by Rotational Guided Growth

**DOI:** 10.3390/jcm13247514

**Published:** 2024-12-10

**Authors:** Michael Zaidman, Naum Simanovsky, Vladimir Goldman, Eden Weisstub

**Affiliations:** Pediatric Orthopedics, Hadassah—Hebrew University Medical Center, Jerusalem 9112001, Israel

**Keywords:** rotational malalignment, rotational guided growth, growing child, 8-plate

## Abstract

**Background**: Femoral torsional malalignment is a common cause of in-toeing and out-toeing in children, often leading to gait disturbances, functional limitations, and increased risk of falls. Traditionally, osteotomy was the only surgical option for correction. A minimally invasive technique known as rotational guided growth (RGG) has recently been introduced to address these malalignments. This study aims to assess the effectiveness of rotational femoral malalignment correction by rotational epiphysiodesis with tension band 8-plates (Orthofix, Verona, Italy). **Methods**: Eleven patients with in-toeing and out-toeing (19 femurs) were treated using RGG with 8-plates. The 8-plates were applied laterally and medially, with screws placed above and below the growth plate of the distal femur, angled obliquely to the long axis of the bone in opposite directions. Changes in foot progression angle (FPA), femoral version, the alteration in the angle between the 8-plates, and the rate of correction were recorded. **Results**: All patients reported functional gait improvement. The FPA was corrected from a mean of 32 degrees to 7 degrees, the femoral version improved from a mean of 60 degrees to 22 degrees. The angle between the 8-plates changed from a mean of 75 degrees to 28 degrees, with a correction rate of 4.1 degrees per month. The average time for correction was 11 months. No complications were observed during the treatment. **Conclusions**: RGG using 8-plates is a novel, minimally invasive surgical technique that effectively corrects rotational femoral deformities and may serve as a preferred alternative to derotational osteotomy in growing patients.

## 1. Introduction

Torsional deformities are relatively common conditions in children [1]. They may arise from post-traumatic malunion, malunion at osteotomy sites, neuromuscular disorders, skeletal dysplasia, congenital and metabolic abnormalities, or have an idiopathic origin [2]. These deformities can lead to gait disturbances and functional limitations. Historically, osteotomy has been the only available corrective option for torsional deformities [3]. Femoral derotational osteotomy presents a risk of under- or over-correction, necessitating a high degree of precision due to the acute nature of the correction. Recently, a minimally invasive technique known as Rotational Guided Growth (RGG) has been introduced to address rotational malalignments [4]. Compared to femoral derotational osteotomy, RGG offers the advantage of a gradual correction process, allowing for greater precision in achieving the desired alignment.

Initial success with rotational changes was demonstrated in animal models, where medial and lateral epiphysiodesis of long bones was used to achieve rotational changes [5,6,7,8,9,10]. Epiphysiodesis was performed obliquely to the long axis of the bone, with opposite directions applied to the medial and lateral sides of the joint, leading to torsional changes during growth. In recent years, this rotational epiphysiodesis technique has been applied for the correction of femoral and tibial torsional deformities in children [11,12].

This study aims to evaluate the effectiveness of femoral rotational malalignment correction using a novel technique of rotational epiphysiodesis with tension band 8-plates. The primary outcomes measured were the correction of foot progression angle (FPA), improvement in gait patterns, and reduction in functional limitations. Secondary outcomes included the correction of femoral version and the change in angle between the 8-plates during the child’s growth.

## 2. Materials and Methods

The study was approved by the Institutional Review Board (IRB approval number 0689-17). We conducted a retrospective review of medical records and radiographs of pediatric patients treated with RGG between 2022 and 2023. The primary indication for RGG to correct rotational malalignment was femoral rotational deformities, which caused gait disturbances and functional limitations in children with at least two years of growth remaining. Functional limitations due to in-toeing were marked by frequent tripping, as the feet tended to catch on each other, resulting in falls. The normal range for foot progression angle (FPA) is typically 5° to 20° of external rotation in children and 5° to 15° in adults [13].

The rotational profile was assessed through physical examination. The femoral version was determined by calculating the difference between internal rotation and external rotation measurements of the hip joint, and by recording the angle at which the greater trochanter was most palpable [14]. Tibial torsion was evaluated using the thigh–foot angle [15]. FPA was measured as the angle formed between the foot’s axis and the line of progression, while the patient walked on a surface leaving footprints [16]. All measurements were performed using a goniometer. Alterations in the angle between the 8-plates were assessed on lateral knee radiographs.

Changes in FPA, femoral version, the angle between the 8-plates, and the rate of correction were documented and analyzed. Any complications during treatment and follow-up were noted. Post-treatment growth continuity was assessed through knee radiographs taken one year after surgery following plate removal.

The Shapiro–Wilk test was first used to assess the normality of data distribution. For non-normal distributions, the Mann–Whitney U test was applied. Statistical analysis was then conducted using a two-tailed paired sample Student’s *t*-test, with statistical significance set at *p* < 0.05 (IBM Corp. Released 2019. IBM SPSS Statistics for Windows, Version 26.0. Armonk, NY, USA: IBM Corp).

### Surgical Procedure

All procedures were performed by staff surgeons with specialized expertise in pediatric orthopedic surgery. Patients were positioned supine on a radiolucent table. Under general anesthesia and with the application of a tourniquet, the 8-plates were inserted with C-arm fluoroscopic guidance. The plates were applied laterally and medially, with screws placed above and below the distal femoral growth plate, angled obliquely to the long axis of the bone in opposing directions (Figure 1). Efforts were made to achieve the largest possible angle between the lateral and medial 8-plates without compromising the growth plate. For external rotation correction, the 8-plate was positioned medially, with the screws placed obliquely from a superior-anterior to an inferior-posterior direction, and laterally from a superior-posterior to an inferior-anterior direction. For internal rotation correction, the plates were positioned in the opposite directions. A skin incision of approximately 2 cm was required. The operative technique for 8-plate application remained unchanged.

Weight-bearing was permitted on the day of surgery, and all patients were discharged either the same day or the following day. Routine follow-up examinations were performed in the outpatient clinic two to three weeks postoperatively, with subsequent visits every three months. The screws and 8-plates were removed once the plates changed orientation from oblique to vertical relative to the long axis of the bone, indicating that the plate had reached its maximum corrective effect (Figure 2).

## 3. Results

A total of 11 patients (19 femurs) were treated with RGG using 8-plates between August 2022 and November 2023 (Table 1). This cohort included five girls and six boys, with a mean age of 11.45 years (range: 8.1–13.9 years). Seven patients had rotational malalignment related to cerebral palsy, with four presenting spastic diplegia and three with hemiplegia. All children with cerebral palsy had a Gross Motor Function Classification System (GMFCS) score of 2, except for one patient who had a score of 3. Four patients were treated for idiopathic rotational malalignment. In our cohort, in-toeing led to functional issues when the FPA reached −20° or more. Additionally, a patient with out-toeing of 45°, also treated with RGG, reported an increased frequency of falls.

The mean follow-up duration was 8.2 months (range: 1–15 months). In five patients, the follow-up period exceeded 14–15 months. All patients with in-toeing reported subjective functional gait improvements, specifically the cessation of tripping and falls, by the end of treatment (Figure 3). Similarly, the patient with out-toeing also noted a cessation of falls following correction of the FPA. The FPA improved significantly from a mean of 32 degrees to 7 degrees (*p* < 0.00001), with a correction rate of 2.2 degrees per month (Table 2 and Table 3). Femoral version, as measured by the difference between internal and external hip rotation, improved from a mean of 60 degrees to 22 degrees (*p* < 0.0001), with a correction rate of 3.36 degrees per month. Using the greater trochanter method, femoral version improved from a mean of 54 degrees to 20 degrees (*p* < 0.0001), with a correction rate of 2.95 degrees per month. The angle between the 8-plates decreased from a mean of 75 degrees to 28 degrees (*p* < 0.0001), corresponding to a correction rate of 4.1 degrees per month. The average time for correction was 11 months.

No complications were observed during the treatment or follow-up period, and no coronal or sagittal plane deformities or recurrences of rotational malalignment were reported. No special rehabilitation program was required in the postoperative period. No growth arrest was observed after 8-plate removal. On control radiographs, an open growth plate was visualized, and the distancing of the screw holes from the growth plate after metaphyseal screw removal further confirmed continued growth plate activity (Figure 4).

## 4. Discussion

The data from this study demonstrates that RGG effectively and safely corrects femoral rotational malalignment and FPA while improving functional gait, with cessation of falls, particularly in neuromuscular patients. The re-orientation of the 8-plates from an oblique to a vertical position served as an objective indicator of rotational changes during RGG.

The 8-plate as an implant device for rotational epiphysiodesis is, to our knowledge, used for the first time in children. The literature on RGG in children is sparse. Only two studies have been published reporting the use of rotational epiphysiodesis techniques in children [11,12]. Metaizeau et al. reported their experience with correcting femoral torsional deformities by guided growth in children using two cannulated screws with a tension band cable for rotational epiphysiodesis, treating 20 knees in 11 children [12]. The primary complication reported was knee stiffness, which resolved completely with physiotherapy and was likely associated with the type of implant used. In our cohort, we believe this issue was avoided due to differences in surgical technique. Specifically, the use of steel cables, as described in their study, may lead to soft tissue entrapment, whereas our technique avoids this risk.

Metaizeau et al. concluded that the method was effective and may constitute an alternative to derotational osteotomy. Paley et al. published his experience using separated halves of a hinge plate connected with Fibertape for RGG in the treatment of femoral and tibial rotational malalignments [11]. All five patients (5 femurs, 3 tibias) demonstrated corrected rotational deformities without recurrence or growth disturbance over a follow-up period of one and a half years. We believe the use of 8-plates for RGG has certain advantages: the implant is rigid, has a long history of use, and the operative technique is simple and well known.

A temporary hemi-epiphysiodesis with tension band 8-plates has become the primary treatment choice for coronal deformity correction when growth remains [17,18,19,20]. The procedure is minimally invasive, has low morbidity, and offers good precision and efficiency. In the past, the only available option for correcting torsional deformities was osteotomy with hardware fixation, a relatively time-consuming procedure. Bilateral surgery would double the operative time and may result in significant blood loss, sometimes necessitating blood transfusion. Strong analgesics are typically required during the postoperative period. A non-weight-bearing or protective weight-bearing period is often needed postoperatively, depending on the type of hardware fixation, and intensive rehabilitation and physiotherapy are needed to restore function, especially in neuromuscular patients [21,22,23].

Following the example of temporary hemi-epiphysiodesis, the introduction of RGG may revolutionize the field of rotational malalignment correction and become the preferred primary treatment protocol for growing patients. In contrast to osteotomy, temporary rotational epiphysiodesis is minimally invasive and performed through an approximately 2 cm incision, potentially reducing surgery and anesthesia duration compared to the osteotomy procedure. There is no need for strong analgesics postoperatively, and minimal blood loss means that no blood transfusion is required. Full weight-bearing and range of motion exercises are allowed immediately after surgery, significantly reducing the rehabilitation process. Additional benefits include more working days for parents and caregivers and increased school attendance for patients.

The primary risk associated with RGG using an 8-plate is growth arrest. This occurs when the plate aligns vertically with the axis of the diaphysis, effectively acting as a growth-inhibiting device on the growth plate. Therefore, close follow-up is crucial, and the plates should be removed promptly once they become vertically aligned.

The limitations of this study include its retrospective design, limited sample size, variations in the underlying causes of rotational malalignments, and the absence of long-term follow-up. Future studies should aim for larger cohorts with long-term follow-up and consider a prospective approach, with an emphasis on stratifying patients by underlying pathology to gain more detailed insights.

## 5. Conclusions

This study confirms that RGG using 8-plates is an effective, minimally invasive method for correcting femoral rotational deformities in children, significantly improving gait, and reducing functional limitations. RGG offers key advantages over osteotomy, including shorter recovery time, reduced pain, and fewer disruptions to patients’ lives.

## Figures and Tables

**Figure 1:**
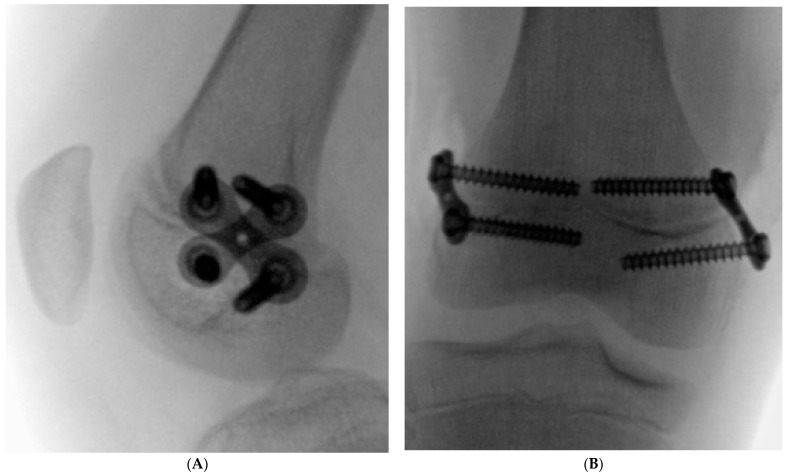
Intraoperative radiographs of an 8-plate positioned obliquely for RGG, with (**A**) showing the lateral view and (**B**) showing the anteroposterior (AP) view.

**Figure 2:**
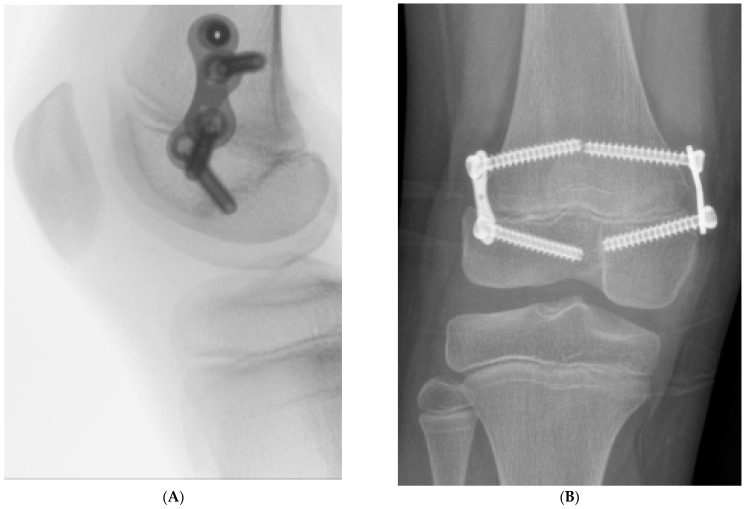
Radiographs showing the 8-plates in a horizontal orientation, with (**A**) representing the lateral view and (**B**) representing the anteroposterior (AP) view, indicating the end of their correction potential in the RGG process.

**Figure 3:**
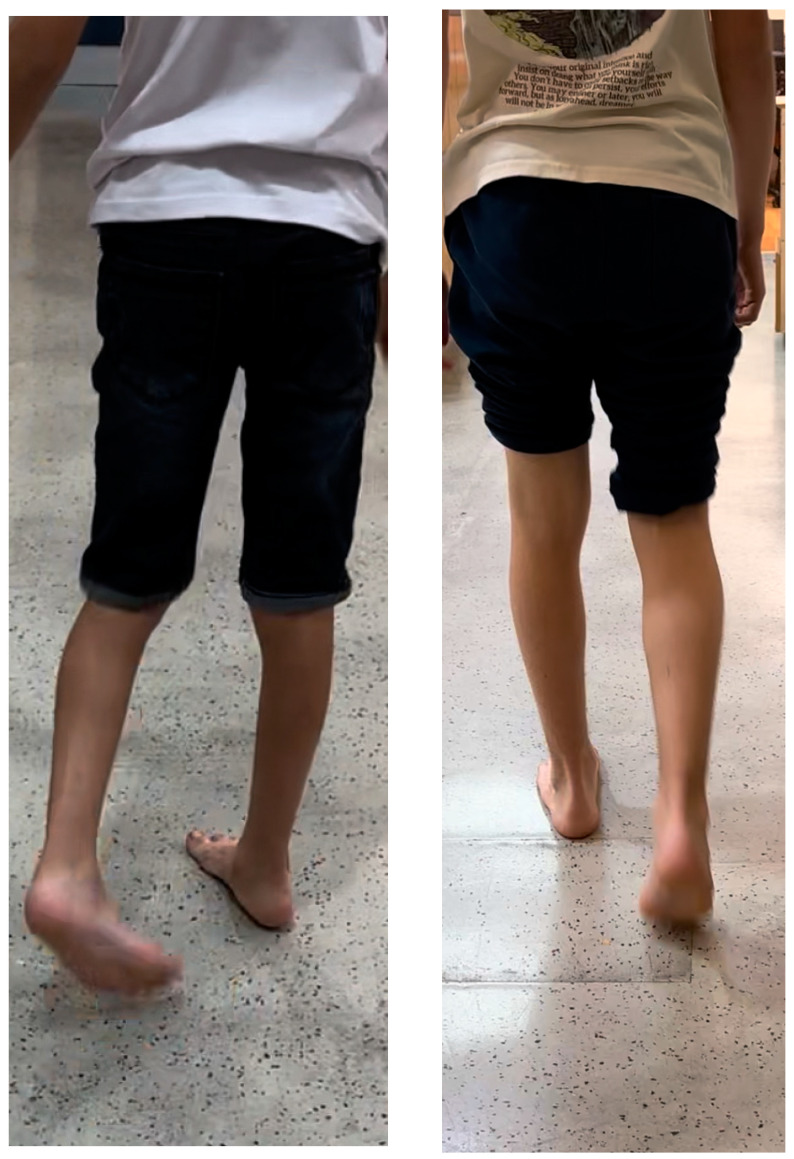
Correction of FPA by RGG: On the **left** side, the patient exhibits noticeable in-toeing. On the **right** side, following RGG, the same patient demonstrates a corrected FPA, showing a successful improvement in gait alignment.

**Figure 4:**
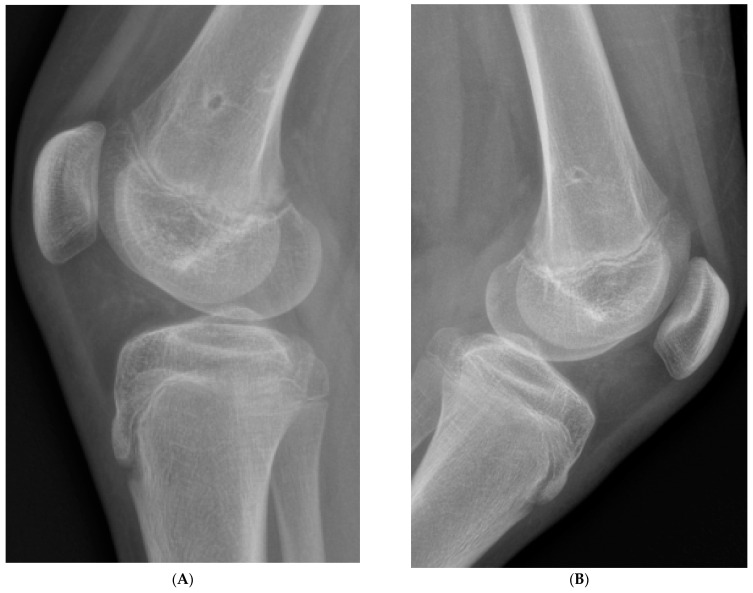
(**A**,**B**) depict lateral radiographs of the knees of two different children, each showing an open growth plate and visible holes from previously placed (and now removed) metaphyseal screws. On follow-up, the holes in both cases have shifted further away from the growth plate, indicating that bone growth remains active in each child.

**Table 1:** Demographic and clinical data.

Patient	Age Years	Gender F/M	Co-Morbidity	Side of RGG	Time Until Correction (Month)	Follow Up (Month)	Functional Gait Improvement
1	13	M	CP, RT HEMI	RT	11.5	15	Yes
2	9.1	F	CP, DIPLEGIA	RT + LT	11.5	14	Yes
3	11.4	F	CP, DIPLEGIA	RT + LT	10	15	Yes
4	12.3	F	CP, RT HEMI	RT	11	14	Yes
5	9.7	M	CP, DIPLEGIA	RT + LT	9	15	Yes
6	10.8	F	CP, DIPLEGIA	RT + LT	14	5	Yes
7	13.9	M	IDIOPATHIC	RT + LT	13	2	Yes
8	12.7	M	CP, RT HEMI	RT	12.5	4	Yes
9	8.1	F	IDIOPATHIC	RT + LT	11	1	Yes
10	12.3	M	IDIOPATHIC	RT + LT	11	4	Yes
11	12.4	M	IDIOPATHIC	RT + LT	11	1	Yes
Mean value	11.45	5:6			11.4	8.2	

RT: right; LT: left; M: male; F: female; CP—Cerebral Palsy; HEMI—hemiplegia.

**Table 2:** Rotational profile and 8-plate angle before and after correction.

Side	FPA Before	FPA After	Hip Version Before	Hip Version After	Hip Version GT Before	Hip Version GT After	8-Plate Angle Before	8-Plate Angle After
RT	−30°	−5°	60°	25°	60°	20°	76°	33°
RT	−40°	−10°	70°	25°	70°	25°	100°	46°
LT	−30°	0°	40°	10°	40°	15°	80°	38°
RT	−30°	−10°	30°	20°	30°	20°	65°	22°
LT	−30°	−10°	30°	20°	30°	20°	85°	38°
RT	−40°	−5°	80°	45°	75°	30°	73°	44°
RT	−40°	−5°	60°	15°	60°	15°	62°	20°
LT	−40°	0°	45°	15°	45°	15°	54°	17°
RT	−40°	−15°	60°	40°	45°	25°	67°	0°
LT	−20°	−5°	50°	40°	40°	30°	61°	6°
RT	−30°	0°	70°	25°	50°	20°	65°	27°
LT	−30°	0°	70°	20°	50°	20°	67°	20°
RT	−30°	0°	65°	35°	60°	20°	81°	43°
RT	−25°	0°	80°	25°	70°	25°	95°	24°
LT	−30°	0°	85°	25°	70°	25°	74°	25°
RT	45°	30°	70°	0°	70°	10°	81°	24°
LT	45°	30°	70°	10°	70°	10°	70°	32°
RT	−30°	−5°	65°	10°	50°	20°	89°	35°
LT	−20°	−5°	50°	15°	50°	20°	87°	42°
Mean	32.4°	7.1°	60.5°	22.1°	53.9°	20.3°	75.4°	28.3°
*p*-value	*p* < 0.00001		*p* < 0.0001		*p* < 0.00001		*p* < 0.0001	

Negative FPA—in-toeing; positive FPA—out-toeing; FPA—foot progression angle; hip version—hip version IR-ER difference; Hip version GT—hip version according to Greater Trochanter measurement; 8-plate angle—angle between medial and lateral 8-plates.

**Table 3:** Correction rates of FPA, hip version, and change in the angle between medial and lateral 8-plates.

	FPA	Hip Version	Hip Version GT	8-Plate Angle
Rate of Correction Degree/month	2.2°	3.36°	2.95°	4.1°

FPA—foot progression angle; Hip version—hip version IR-ER difference; Hip version GT—hip version according to Greater Trochanter measurement; 8-plate angle—angle between medial and lateral 8-plates.

## Data Availability

The data presented in this study are available on request from the corresponding author. The data are not publicly available due to data access limitation imposed by the institutional ethics committee.

## References

[1] Lincoln T.L., Suen P.W. (2003). Common rotational variations in children. J. Am. Acad. Orthop. Surgeons.

[2] Celestre P.C., Bowen R.E. (2009). Correction of angular deformities in children. Curr. Orthop. Practice.

[3] Dreher T., Wolf S.I., Heitzmann D., Swartman B., Schuster W., Gantz S., Hagmann S., Döderlein L., Braatz F. (2012). Long-term outcome of femoral derotation osteotomy in children with spastic diplegia. Gait Posture.

[4] Sawiris Y.A., Abo-Seif S., Aly A.S. (2018). The Using of ‘Guided Growth’ for Correction of Coronal Deformities around the Knee in Skeletally Immature Children (Systematic review and Metaanalysis). Med. Clin. Reviews.

[5] Arami A., Bar-On E., Herman A., Velkes S., Heller S. (2013). Guiding femoral rotational growth in an animal model. J. Bone Jt. Surg..

[6] Sevil-Kilimci F., Cobanoglu M., Ocal M.K., Korkmaz D., Cullu E. (2019). Effects of Tibial Rotational-guided Growth on the Geometries of Tibial Plateaus and Menisci in Rabbits. J. Pediatr. Orthop..

[7] Lazarus D.E., Farnsworth C.L., Jeffords M.E., Marino N., Hallare J., Edmonds E.W. (2018). Torsional Growth Modulation of Long Bones by Oblique Plating in a Rabbit Model. J. Pediatr. Orthop..

[8] Cobanoglu M., Cullu E., Kilimci F.S., Ocal M.K., Yaygingul R. (2016). Rotational deformities of the long bones can be corrected with rotationally guided growth during the growth phase. Acta Orthop..

[9] Abood A.A., Rölfing J.D., Halloum A., Ringgaard S., Byskov J.S., Kold S., Rahbek O. (2024). An Innovative Plate Concept for Rotational Guided Growth: A Porcine Pilot Study. Cureus.

[10] Martel G.A., Holmes L., Sobrado G., Gabriela A., Eduardo S., Paley D., Praglia F., Arguello G., Arellano E., Flores G.R. (2018). Rotational-guided growth. J. Limb Lengthening Reconstr..

[11] Paley D., Shannon C. (2022). Rotational Guided Growth: A Preliminary Study of Its Use in Children. Children.

[12] Metaizeau J.D., Denis D., Louis D. (2019). New femoral derotation technique based on guided growth in children. Orthop. Traumatol. Surg. Res..

[13] Perry J., Burnfield J.M., Cabico L.M. (2010). Gait Analysis: Normal and Pathological Function. J. Sports Sci. Med..

[14] Davids J.R., Benfanti P., Blackhurst D.W., Allen B.L. (2002). Assessment of Femoral Anteversion in Children with Cerebral Palsy: Accuracy of the Trochanteric Prominence Angle Test. J. Pediatr. Orthop..

[15] Staheli L.T., Corbett M., Wyss C., King H. (1985). Lower-extremity rotational problems in children. Normal values to guide management. J. Bone Jt. Surg..

[16] Lösel S., Burgess-Milliron M.J., Micheli L.J., Edington C.J. (1996). A Simplified Technique for Determining Foot Progression Angle in Children 4 to 16 Years of Age. J. Pediatr. Orthop..

[17] Farr S., Alrabai H.M., Meizer E., Ganger R., Radler C. (2018). Rebound of Frontal Plane Malalignment After Tension Band Plating. J. Pediatr. Orthop..

[18] Gottliebsen M., Møller-Madsen B., Stødkilde-Jørgensen H., Rahbek O. (2013). Controlled longitudinal bone growth by temporary tension band plating: An experimental study. Bone Jt. J..

[19] Stevens P.M. (2007). Guided growth for angular correction: A preliminary series using a tension band plate. J. Pediatr. Orthop..

[20] Ballal M.S., Bruce C.E., Nayagam S. (2010). Correcting genu varum and genu valgum in children by guided growth: Temporary hemiepiphysiodesis using tension band plates. J. Bone Jt. Surg..

[21] Schenke M., Dickschas J., Simon M., Strecker W. (2018). Corrective osteotomies of the lower limb show a low intra- and perioperative complication rate-an analysis of 1003 patients. Knee Surg. Sports Traumatol. Arthrosc..

[22] Kim H., Aiona M., Sussman M. (2005). Recurrence after femoral derotational osteotomy in cerebral palsy. J. Pediatr. Orthop..

[23] Mycoskie P.J. (1981). Complications of osteotomies about the knee in children. Orthopedics.

